# Using respiratory rate and thoracic movement to assess respiratory insufficiency in amyotrophic lateral sclerosis: a preliminary study

**DOI:** 10.1186/1472-684X-11-26

**Published:** 2012-12-27

**Authors:** Waltteri Siirala, Tarja Saaresranta, Arno Vuori, Sanna Salanterä, Klaus T Olkkola, Riku Aantaa

**Affiliations:** 1 Department of Anaesthesiology, Intensive Care, Emergency Care and Pain Medicine, Turku University Hospital and University of Turku, Turku, Finland; 2 Department of Pulmonary Diseases, Turku University Hospital, Turku, Finland; 3 Department of Nursing Science, University of Turku, Turku, Finland

**Keywords:** Amyotrophic lateral sclerosis, Hypoventilation, Non-invasive ventilation

## Abstract

**Background:**

Hypoventilation due to respiratory insufficiency is the most common cause of death in amyotrophic lateral sclerosis (ALS) and non-invasive ventilation (NIV) can be used as a palliative treatment. The current guidelines recommend performing spirometry, and recording nocturnal oxyhemoglobin saturation and arterial blood gas analysis to assess the severity of the hypoventilation. We examined whether the respiratory rate and thoracic movement were reliable preliminary clinical signs in the development of respiratory insufficiency in patients with ALS.

**Methods:**

We measured the respiratory rate and thoracic movement, performed respiratory function tests and blood gas analysis, and recorded subjective hypoventilation symptoms in 42 ALS patients over a 7-year period. We recommended NIV if the patient presented with hypoventilation matching the current guidelines. We divided patients retrospectively into two groups: those to whom NIV was recommended within 6 months of the diagnosis (Group 1) and those to whom NIV was recommended 6 months after the diagnosis (Group 2). We used the Mann Whitney U test for comparisons between the two groups.

**Results:**

The mean partial pressure of arterial carbon dioxide in the morning in Group 1 was 6.3 (95% confidence interval 5.6–6.9) kPa and in Group 2 5.3 (5.0–5.6) kPa (p = 0.007). The mean respiratory rate at the time of diagnosis in Group 1 was 21 (18–24) breaths per minute and 16 (14–18) breaths per minute in Group 2 (p = 0.005). The mean thoracic movement was 2.9 (2.2–3.6) cm in Group 1 and 4.0 (3.4–4.8) cm in Group 2 (p = 0.01). We observed no other differences between the groups.

**Conclusions:**

Patients who received NIV within six months of the diagnosis of ALS had higher respiratory rates and smaller thoracic movement compared with patients who received NIV later. Further studies with larger numbers of patients are needed to establish if these measurements can be used as a marker of hypoventilation in ALS.

## Background

Amyotrophic lateral sclerosis (ALS) is a form of degenerative motor neuron disease of unknown etiology. The disease is characterized by progressive muscle weakness and atrophy throughout the body [1-3]. The prevalence is 4–8 in 100 000 and the annual incidence is 1–2 in 100 000 [4]. Prognosis is poor with a median survival from the onset of symptoms of 2–4 years [5,6]. Despite extensive effort, no curative treatment is available and riluzole (a tetrodotoxin-sensitive sodium channel blocker) is the only drug that can slow the progression of the disease [7,8]. Therefore, treatment following the diagnosis is palliative [9,10].

Hypoventilation due to respiratory insufficiency is the most common cause of death in patients with advanced ALS [3,5]. Non-invasive ventilation (NIV) has been recommended for ALS patients when hypoventilation occurs because it relieves dyspnoea, increases the quality of life, and may prolong survival in late stage ALS patients [11-13]. The current guidelines recommend beginning NIV if the patient presents with dyspnoea, orthopnoea, disturbed sleep, tachypnoea, nocturnal desaturation < 90%, increased morning carbon dioxide partial pressure (pCO_2_) > 6 kPa, decreased sniff nasal pressure < 40 cmH_2_O, decreased maximum inspiratory mouth pressure (MIP) < 60 cmH_2_O, or decreased forced vital capacity (FVC) < 80% [9,10,14]. However, there is great international variation in the use of different diagnostic tests prior to initiation of NIV.

The onset of muscle weakness varies between patients and symptoms may appear first in the limbs or they may start from the bulbar area leading to dysphagia and speech difficulties [3]. This wide variability in the clinical course of ALS can be challenging for the clinician because the progression of respiratory insufficiency also greatly varies among these patients [1,3]. Because of this variability, the current guidelines recommend a clinical visit every 2–3 months [9,10]. Although nocturnal desaturation and carbon dioxide tension can be recorded noninvasively, it is often not possible to perform these measurements outside the hospital because of lack of devices and health care providers to assist the patient. The measurement of increased morning pCO_2_ requires blood sampling by a health care professional. In addition, spirometric measurements require good facial function, as patients have to hold their lips tightly around the mouthpiece of the spirometer. In ALS patients with severe bulbar dysfunction, this measurement may be unreliable [10,15]. We therefore wished to determine if we could use respiratory rate and thoracic movement as preliminary clinical signs in the development of respiratory insufficiency.

## Methods

### Design

**Figure 1:**
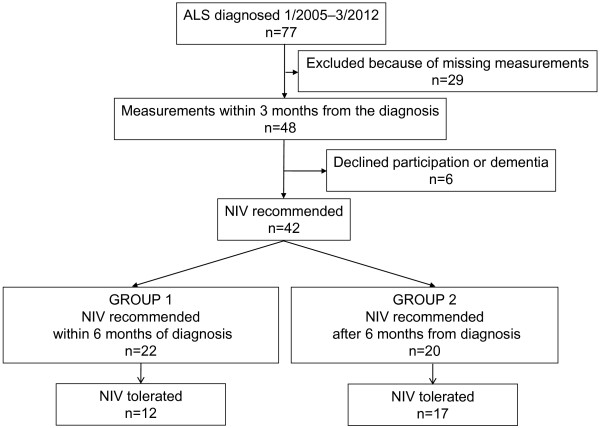
Study flow chart.

This study was a retrospective register study. According to Finnish legislation, patient consent is not required for register studies in Finland. The study protocol was approved by the ethics committee of the Hospital District of South-West Finland. The data were collected as part of routine measurements in patients diagnosed with ALS at the Turku University Hospital during January 2005 to March 2012. A total of 77 patients fulfilled the El Escorial World Federation criteria for ALS [16]. NIV treatment and other palliative treatments were offered to all the patients. The respiratory measurements were obtained within three months of the diagnosis in only 42 patients who were included in the per-protocol analysis of the data. Twenty-nine patients were excluded because respiratory measurements were not performed within the three-month period from the diagnosis. The three-month time window was assumed to reflect the respiratory function of the patients at the time of the ALS diagnosis. We excluded an additional three patients because of lack of cooperation (frontotemporal dementia) and three patients declined follow-up for NIV. Although NIV was recommended for 42 patients, 29 of these 42 patients were able to use NIV. Based on earlier studies [5,6] in which the course of the disease was rapid, we divided these 42 patients retrospectively into two groups: those for whom NIV was recommended within 6 months from the diagnosis (Group 1; n = 22) and those for whom NIV was recommended after 6 months from the diagnosis (Group 2; n = 20) (Figure 1). Before the NIV trial, we asked the patients if they were willing to start the treatment. Each patient made the final decision whether to begin NIV.

### Data collection

The patients were referred to the Department of Pulmonary Diseases, following diagnosis of ALS by a neurologist. An experienced respiratory physiotherapist performed the respiratory function tests. The respiratory rate was assessed before any other measurements with the patient awake, in a supine position and following a one-hour rest. A specialized nurse observed and calculated the respiratory rate during one minute. We measured forced vital capacity (FVC) and forced expiratory volume exhaled in 1s (FEV 1) using a hand-held MicroPlus® spirometer (Cardinal Health, Chatham, UK), and expressed each as a fraction of the predicted values in per cent [17]. Respiratory muscle tests included assessments of peak cough flow (PCF), maximum inspiratory mouth pressure (MIP), maximum expiratory mouth pressure (MEP), and sniff nasal pressure (SNP), all measured with MicroRPM® (Cardinal Health, Chatham, UK). We performed each measurement three times with the patient in a sitting position and submitted the best value for the analyses. We measured the thoracic movement at the mammillary level with the patient in a sitting position and recorded the difference in the thoracic circumference between the maximal inspiration and expiration. The respiratory rate was expressed as breaths per min (BPM) at rest. An arterial blood gas sample was drawn from the radial artery in the morning just after waking and with the patient in a supine position.

We assessed the subjective symptoms of hypoventilation using a set of visual analogue scales (VAS) using a 10 cm long line with opposite extremes at each end, where 0 indicated no symptoms and 10 indicated the worst imaginable symptoms; assessing dyspnoea, cough weakness, sleep disturbances, morning headaches, and daytime sleepiness. If a patient was not able to tick the line on the VAS because of impaired motor function of his/her hand, the physiotherapist marked the patient’s response.

### Ventilatory support

NIV was provided using a pressure-targeted ventilator (VPAP III ST®, ResMed, Bella Vista, Australia). Both a pulmonologist and an anaesthesiologist assessed the need for NIV. The primary criteria for recommendation for an NIV trial were dyspnoea at rest, increased pCO_2_ > 5.5 kPa or decreased pO_2_ < 10 kPa in the morning arterial blood gases or decreased FVC under 50% of the predicted value. The secondary criteria for recommendation for an NIV trial were MIP < 60 cmH_2_O, or SNP < 40 cmH_2_O. We interpreted a VAS score > 5 for dyspnoea as an indication to recommend an NIV trial. The remainders of the measurements were used as supportive criteria to recommend an NIV trial.

### Statistical analyses

The results are shown as mean with a 95% confidence interval unless otherwise stated. We used the Mann Whitney U test for comparisons between the two groups at the time of the diagnosis. P values < 0.05 were considered statistically significant. We used GraphPad Prism Software, Version 5.00 (San Diego, California, USA) for the statistical calculations.

## Results

The records for 42 patients with ALS (20 male, 22 female) were adequate for analysis. The characteristics of the patients are shown in Table 1. The age and the duration of the symptoms before the diagnosis of ALS did not differ between the groups.

**Table 1:** Patient characteristics

	**Group 1 (n=22)**		**Group 2 (n=20)**		
	**Median**	**Range**	**Median**	**Range**	**p**
Age at diagnosis (years)	69	49–83	69	54–85	0.9
Duration from the first symptoms to diagnosis (months)	11	1–48	13	3–60	0.8
Duration from the diagnosis to initiation of NIV (months)	2	0–5	12	6–29	< 0.001

The difference between the groups was assessed using the Mann Whitney U test.

NIV = non-invasive ventilation.

The mean pCO_2_ and pO_2_ in the morning arterial blood gas samples at the time of diagnosis in Group 1 were 6.3 (5.6–6.9) and 9.8 (9.3–10.4) kPa, respectively. The mean pCO_2_ and pO_2_ at the time of diagnosis in Group 2 were 5.3 (5.0–5.6) and 10.7 (9.4–12) kPa, respectively. The mean pCO_2_ was significantly higher in Group 1 (p = 0.007) whereas the values for mean pO_2_ did not differ between the two groups (p = 0.4). The mean respiratory rate at the time of diagnosis in Group 1 was 21 (18–24) BPM and 16 (14–18) BPM in Group 2 (p = 0.005). The mean thoracic movement at the time of diagnosis in Group 1 was 2.9 (2.2–3.6) cm and 4.0 (3.4–4.8) cm in Group 2 (p = 0.01). We observed no other differences between the two groups (Table 2).

**Table 2:** Respiratory function tests, blood-gas analysis and subjective symptoms at the time of diagnosis

	**Group 1**		**Group 2**		
**Measurement**	**Mean (95% CI)**	**n**	**Mean (95% CI)**	**n**	p*
*Lung function tests*					
FVC (%)	58 (44–72)	20	69 (58–74)	20	0.4
FEV 1/s (%)	59 (46–72)	20	69 (61–72)	20	0.2
*Respiratory muscle force*					
MIP (cmH_2_O)	29 (20–39)	21	48 (30– 65)	20	0.07
MEP (cmH_2_O)	40 (28–52)	21	60 (42–78)	20	0.07
SNP (cmH_2_O)	24 (9–40)	18	31 (21–41)	18	0.1
PCF (l/min)	240 (180–300)	18	310 (250–370)	20	0.1
*Severity of hypoventilation symptoms*					
Dyspnoea	4 (3–6)	21	2 (1–3)	20	0.05
Cough weakness	3 (2–5)	21	3 (1–4)	20	0.4
Sleep disturbance	5 (3–6)	21	3 (2–3)	20	0.05
Morning headaches	2 (0–3)	21	1 (0–2)	20	0.7
Daytime sleepiness	4 (3–6)	21	4 (2–5)	20	0.4
*Thoracic movement* (cm)	2.9 (2.2–3.6)	22	4.0 (3.4–4.8)	20	0.01
*Respiratory rate* (breaths/min)	21 (18–24)	22	16 (14–18)	20	0.005
*Arterial blood-gas analysis*					
pCO_2_ (kPa)	6.3 (5.6–6.9)	17	5.3 (5.0–5.6)	12	0.007
pO_2_ (kPa)	9.8 (9.3–10.4)	17	10.7 (9.4–12)	12	0.4

*Determined using the Mann Whitney U test.

n = number of patients completing the measurement; CI = confidence interval; FVC = forced vital capacity expressed as % from the reference values; FEV 1 = forced expiratory volume exhaled in one second and expressed as % from the reference values; MIP = maximal inspiratory mouth pressure; MEP = maximal expiratory mouth pressure; SNP = sniff nasal pressure; PCF = peak cough flow. All measurements were performed three times in a sitting position and the best values were recorded.

The severity of hypoventilation symptoms was assessed using a visual analogue scale (VAS) in which 0 represented no symptoms and 10 represented the worst symptoms that the patients could imagine. Thoracic movement was measured using a measuring tape and recorded as the difference in the thoracic circumference during maximal inspiration and expiration at the mammillary level.

## Discussion

This was a retrospective study to clarify if we could use the respiratory rate and thoracic movement as preliminary clinical signs for the development of significant respiratory insufficiency in ALS patients. The main finding in our study was that patients who received NIV within six months of the diagnosis of ALS had higher respiratory rates and smaller thoracic movement at diagnosis compared with patients who received NIV later.

Among healthy subjects, a respiratory rate under 15 BPM allows a physiologically optimal level of work of breathing [18,19]. In advanced ALS, thoracic compliance is usually decreased, resulting in decreased tidal volume [20], increased respiratory rate and work of breathing [18,19]. NIV can help patients compensate for the decreased thoracic compliance and thus decrease the respiratory frequency and the work of breathing [20,21]. Although the retrospective study design and small number of patients did not allow the calculation of positive and negative predictive values and cut-off values for respiratory rate and thoracic movement in the assessment of respiratory insufficiency in ALS, it appears that NIV was initiated within six months from diagnosis for those patients who had a respiratory rate at least of 20 BPM at the moment of diagnosis.

Another result of interest was that we failed to show any difference in dyspnoea between the two study groups (Table 2). The patients scored only mild symptoms of dyspnoea and morning headaches at the moment of diagnosis in both groups. The same was true for sleep disturbances and daytime sleepiness. In a previous study with 36 ALS patients, respiratory complaints did not occur until the vital capacity, MIP, or MEP were severely impaired [22]. In that study, the strongest correlation with dyspnoea was a decline in vital capacity. The authors suggested that a small reduction in vital capacity does not cause any symptoms except in heavy exercise. The patients decrease their physical activity because of skeletal muscle weakness, and thus may not feel dyspnoeic during their daily activities unless more strenuous exercise is required. Our results are similar as our patients complained of very little dyspnoea at the time of diagnosis. Nevertheless, as our patients were aware of the possibility of initiating NIV, they might have been reluctant to disclose the severity of their symptoms and intentionally underestimated their response in our VAS query. A larger sample size might show a difference in dyspnoea.

FVC and FEV 1 are commonly used to assess pulmonary function in lung diseases [23] and in ALS patients. Previously, FVC, SNP, MIP and MEP were determined in 16 ALS patients monthly over a period of 18 ± 10 months [24]. The SNP, MIP and MEP were severely reduced even though the FVC remained normal. In addition, the measurement of MIP and MEP was difficult in these advanced ALS patients because they had difficulties tightly holding the mouthpiece of the recording device. This led to air leaks and reduced the values of MIP and MEP. A reduced SNP of under 40 cmH_2_O has been suggested as the most sensitive and easiest test to perform in ALS patients [9,10,24]. However, although we found that the SNP was severely reduced in both groups, only those with an increased respiratory rate in Group 1 were ready to start NIV (Table 2).

Our study has limitations. First, the retrospective design compared the respiratory measurements between two different patient groups at one single time point. Thus, we cannot draw definitive conclusions for the cut-off values for respiratory rate or thoracic movement to indicate the need for NIV. Further studies are needed. In addition, the only inclusion criterion was the respiratory assessment within three months from diagnosis. Thus, we did not evaluate other pulmonary or heart diseases which might have biased the results. Second, the ideal is a randomized prospective trial in which the initiation of NIV is blinded and the subjects are observed from an asymptomatic stage until severe hypoventilation occurs. However, because NIV is established in palliative care in ALS [9,10], ethical aspects have to be considered in randomized study designs, especially if the initiation of NIV is somehow blinded. Moreover, the diagnosis of ALS is often delayed and the patients often have reduced ventilation capacity at the time of diagnosis [22,25]. We saw this in our patients as the median time from the first symptoms until diagnosis ranged from 11 to 13 months (Table 1). During this time, the patients’ symptoms were evaluated at the primary care level or not at all. Thus, when we met the patients, half already showed moderately to severely reduced FVC (60%), SNP (24 cmH_2_O), MIP (29 cmH_2_O), or MEP (41 cmH_2_O) and NIV was recommended based on a single measurement without follow-up. Third, we dealt with severely ill patients, whose NIV initiation was based on both the clinical measurements and the patient’s own desire. The fact that NIV was based on the patient’s own desire may have biased the answers in the hypoventilation questionnaire if the patients reported no or only mild symptoms of hypoventilation even if they had severely reduced respiratory measurements.

## Conclusions

The use of NIV as palliative treatment has previously been shown to relieve symptoms as well as to improve survival in ALS patients [11-13]. However, the optimal timing for initiating NIV is not yet well established. Most of the available methods used to evaluate the degree of hypoventilation and the need for NIV require well-trained health care professional and special devices. In contrast, assessing resting respiratory rate or thoracic movement can be performed in a patient’s home by primary care nursing staff and could be a feasible screening tool for detecting hypoventilation. However, further studies are needed to define the positive and negative predictive values and cut-off values for respiratory rate and thoracic movement in the assessment of respiratory insufficiency in ALS.

## Abbreviations

ALS: Amyotrophic lateral sclerosis; NIV: Non-invasive ventilation; FVC: Forced vital capacity; FEV 1: Forced expiratory volume exhaled in 1s; PCF: Peak cough flow; MIP: Maximum inspiratory mouth pressure; MEP: Maximum expiratory mouth pressure; SNP: Sniff nasal pressure.

## Competing interests

The authors declare that they have no competing interests.

## Authors’ contributions

WS, TS, AV, KO and RA designed the study. WS, TS and AV collected the data. WS and TS were responsible for the interpretation of the data. WS, TS and SS prepared the manuscript. All authors participated in critical revision of the article for important intellectual content, and approved the final version of the article.

## Pre-publication history

The pre-publication history for this paper can be accessed here:

http://www.biomedcentral.com/1472-684X/11/26/prepub
